# Polymorphisms of *MTHFR* and *TYMS* predict capecitabine-induced hand-foot syndrome in patients with metastatic breast cancer

**DOI:** 10.1186/s40880-019-0399-z

**Published:** 2019-10-11

**Authors:** Shaoyan Lin, Jian Yue, Xiuwen Guan, Peng Yuan, Jiayu Wang, Yang Luo, Ying Fan, Ruigang Cai, Qiao Li, Shanshan Chen, Pin Zhang, Qing Li, Fei Ma, Binghe Xu

**Affiliations:** 1 Department of Medical Oncology, National Cancer Center/National Clinical Research Center for Cancer/Cancer Hospital, Chinese Academy of Medical Sciences & Peking Union Medical College, No.17, Panjiayuan Nanli, Chaoyang District, Beijing, 100021 P. R. China; 2 Department of VIP Medical Services, National Cancer Center/National Clinical Research Center for Cancer/Cancer Hospital, Chinese Academy of Medical Sciences & Peking Union Medical College, Beijing, 100021 P. R. China

**Keywords:** Capecitabine, Polymorphism, Metastatic breast cancer, Hand-foot syndrome, *TYMS*, *MTHFR*

## Abstract

**Background:**

Breast cancer is a global problem, and a large number of new cases are diagnosed every year. Capecitabine is effective in patients with metastatic breast cancer (MBC). Hand-foot syndrome (HFS) is a common adverse effect of capecitabine. In this study, we investigated the association between single nucleotide polymorphisms (SNPs) in genes involved in capecitabine metabolism pathways and capecitabine-induced HFS in Chinese patients with MBC to identify some predictive genetic biomarkers.

**Methods:**

We selected 3 genes involved in capecitabine metabolism and screened genetic variants in these target genes. We genotyped a total of 22 SNPs in the thymidylate synthase gene (*TYMS*), the methylene tetrahydrofolate reductase gene (*MTHFR*), and the ribonucleotide reductase M1 gene (*RRM1*) in 342 MBC patients treated with capecitabine-based chemotherapy. The genotype distributions of each SNP in patients with and without HFS were assessed using Pearson’s χ^2^ test, and the relationship between HFS and genotypes of SNPs was determined using logistic regression analysis. The association between SNPs and their corresponding gene expression was analyzed using the Blood expression quantitative trait loci (eQTL) browser online tools.

**Results:**

We found 4 positive sites for HFS in the *TYMS* and *MTHFR* genes: *TYMS* rs2606241 (*P *= 0.022), *TYMS* rs2853741 (*P *= 0.019), *MTHFR* rs3737964 (*P *= 0.029), and *MTHFR* rs4846048 (*P *= 0.030). Logistic regression analyses showed that the genotype AG of *MTHFR* rs3737964 [odds ratio (OR) = 0.54, 95% confidence interval (CI) 0.31–0.97, *P* = 0.038] and *MTHFR* rs4846048 (OR = 0.54, 95% CI 0.30–0.98, *P* = 0.042) were protective factors of HFS, whereas the genotype CT of *TYMS* rs2853741 (OR = 2.25, 95% CI 1.31–3.87, *P* = 0.012) increased the risk of HFS. The association between the genotype GT of *TYMS* rs2606241 (OR = 1.27, 95% CI 0.73–2.23, *P* = 0.012) and HFS was uncertain. Further eQTL analyses confirmed that the alleles of rs3737964 and rs4846048 affected the gene expression levels of *MTHFR* in *cis*.

**Conclusions:**

We have identified four potentially useful pharmacogenetic markers, *TYMS* rs2606241, *TYMS* rs2853741, *MTHFR* rs3737964, and *MTHFR* rs4846048 to predict capecitabine-induced HFS in MBC patients.

## Background

Breast cancer is a global problem, and 1.7 million new cases are diagnosed per year [1]. Approximately 6%–10% of breast cancer patients present metastatic disease when initially diagnosed, and over 30% of patients with non-metastatic disease will relapse [2]. Capecitabine (N4-pentyloxycarbonyl-5′-deoxy-5-fluorocytidine) has been widely used for the treatments of breast [3, 4], colon [5], and gastric cancers [6], and has also been considered as an option for hepatocellular carcinoma [7] and rectal cancer [8]. Capecitabine is often administrated as second-line monotherapy for metastatic breast cancer (MBC) patients whose disease is resistant to anthracycline, taxane, or both [9]. The common capecitabine-induced adverse events include hand-foot syndrome (HFS), increased bilirubin, diarrhea, stomatitis, nausea, neutropenia, and cardiotoxicity [10, 11]. Despite not life-threatening, HFS, characterized by tenderness, redness, and swelling of palms and soles, can be very debilitating and impair the quality of life. Although HFS is manageable, if it is not handled well, it can deteriorate rapidly and lead to treatment interruptions which may affect the treatment efficacy [12].

Capecitabine, a novel oral fluoropyrimidine carbamate, may be converted to 5-fluorouracil (5-FU) selectively in tumors through a cascade of different enzymes [13]. Thymidylate synthase (TYMS), methylene tetrahydrofolate reductase (MTHFR), and ribonucleotide reductase M1 (RRM1) are involved in capecitabine metabolism pathways. The metabolic pathways by which 5-FU and the prodrug capecitabine are converted to active nucleotide analogues are described in details [14]. TYMS catalyzes the conversion of deoxyuridine monophosphate (dUMP) to deoxythymidine monophosphate (dTMP), MTHFR converts 5-10 methylenetetrahydrofolate (5-10 MTHF) into 5-methylenetetrahydrofolate (5-MeTHF) [14], and RRM1 is involved in ribonucleotide reductase reaction converting fluorouridine diphosphate (FUDP) to fluorodeosyuridine diphosphate (FdUDP) [15]. Single nucleotide polymorphisms (SNPs) are used in researches comparing genotype frequencies and copy number variations between cases and controls to identify new cancer-susceptible genes and potential markers predicting therapy response and drug resistance. Any failure in the metabolism system, with a special distinction of SNP genotypes present in the drug-metabolic genes, might result in drug resistance or therapy toxicity.

The availability of tools for predicting toxicity would allow physicians to choose proper treatment regimens to elicit a positive response while keeping adverse effects under acceptable levels. There have been several studies about the biomarkers predicting toxicity of capecitabine, which have been mainly focused on a limited number of known candidates, such as dihydropyrimidine dehydrogenase gene (*DPYD*) [16, 17] and cytidine deaminase gene (*CDD*) [18], based on white patient population. However, until recently, only a limited number of SNPs in *MTHFR* [19], *TYMS* [17], and no SNPs in *RRM1* genes associated with HFS have been identified. Accordingly, we genotyped 22 SNPs in *MTHFR, TYMS*, and *RRM1* genes involved in capecitabine metabolism pathways and combined the medical data with the experimental results in hope of seeking potential genetic markers, which may help identify candidate patients suitable for capecitabine treatment, thus promoting the benefit from capecitabine and avoiding severe adverse effects.

## Patients and methods

### Patient selection

The study was conducted on female MBC patients admitted to the National Cancer Center/National Clinical Research Center for Cancer/Cancer Hospital, Chinese Academy of Medical Sciences & Peking Union Medical College (Beijing, China) between January 2010 and September 2012. All patients received capecitabine-based therapy (capecitabine: 1000 mg/m^2^ orally twice daily, on days 1–14), mostly in combination with docetaxel (75 mg/m^2^, 1-h intravenous infusion on day 1) and vinorelbine (25 mg/m^2^ for 20-min intravenous infusion or 60 mg/m^2^ orally on days 1 and 8) every 3 weeks as one cycle. The inclusion criteria were as follows: female patients with MBC confirmed by pathological or cytological techniques; patients without other malignant cancers; patients eligible for capecitabine-based therapy; complete medical records including age at diagnosis, tumor size, lymph node status, stage, estrogen receptor (ER) status, progesterone receptor (PR) status, human epidermal growth factor receptor 2 (HER2) status, menstrual situation, and previous locoregional or systemic therapy; and complete follow-up data. Regular outpatient or telephone follow-ups were carried out, and the last follow-up was March 1, 2014. Follow-up examinations included computed tomography (CT) of metastases, breast ultrasounds, and tumor marker examination. Treatment responses were evaluated every two cycles of treatment (21 days per cycle) according to Response Evaluation Criteria In Solid Tumors (RECIST) 1.0.

### SNP selection

We selected genes possibly related to toxicity of capecitabine-based chemotherapy in MBC patients, according to the metabolism pathway of capecitabine. Three candidate genes were selected: *TYMS*, *MTHFR*, and *RRM1*. A total of 22 SNPs from the public database (http://hapmap.ncbi.nlm.nih.gov/) and the 1000 Genomes Project database (http://www.1000genomes.org) [20] in 3 key genes in the Chinese Han population were genotyped. All loci were in the balance of Hardy–Weinberg (*P *> 0.05), with minor allele frequency greater than 0.05.

### DNA extraction

We collected 2 mL heparin-anticoagulated blood samples from all participants, and extracted DNA from peripheral blood leucocytes by the phenol–chloroform method using a blood DNA kit (BioTeke Corporation, Beijing, China). The blood samples were stored at − 80 °C, then added to a centrifuge tube after melting at room temperature. The tube was tightly capped and centrifuged for 15 min at 5000×*g*. The supernatant was discarded, and the pellet was suspended with lysis buffer containing 10 mmol/L Tris–HCl (pH 8.0), 0.1 mol/L ethylene diamine tetraacetic acid, 20 μg/mL RNA enzyme, and 0.5% sodium-dodecyl sulphate for 1 h at 37 °C. The cellular lysates were digested overnight at 37 °C with proteinase K (100 μg/mL). After digestion, samples were subsequently blended with a same volume of Tris–HCl-satured phenol (pH 7.4) and centrifuged for 15 min at 8000×*g*.

The aqueous phase was added with a same volume of phenol–chloroform (1:1) in a new tube and centrifuged for 15 min at 8000×*g*. The aqueous phase was collected again in a new tube and precipitated with a 10% volume of ammonium acetate (10 mol/L) and a two times volume of absolute ethanol at − 20 °C. The pellet was rinsed with 75% ethanol twice and resuspended with appropriate TE buffer. The concentration of extracted DNA was assessed using a spectrophotometer. According to Sequenom, the concentration should be higher than 10 ng/μL, and the A_260/280_ ratio between 1.8 and 2.0. The extracted DNA samples were stored in 1.5-mL EP tubes at − 80 °C.

### SNP genotyping

**Fig. 1:**
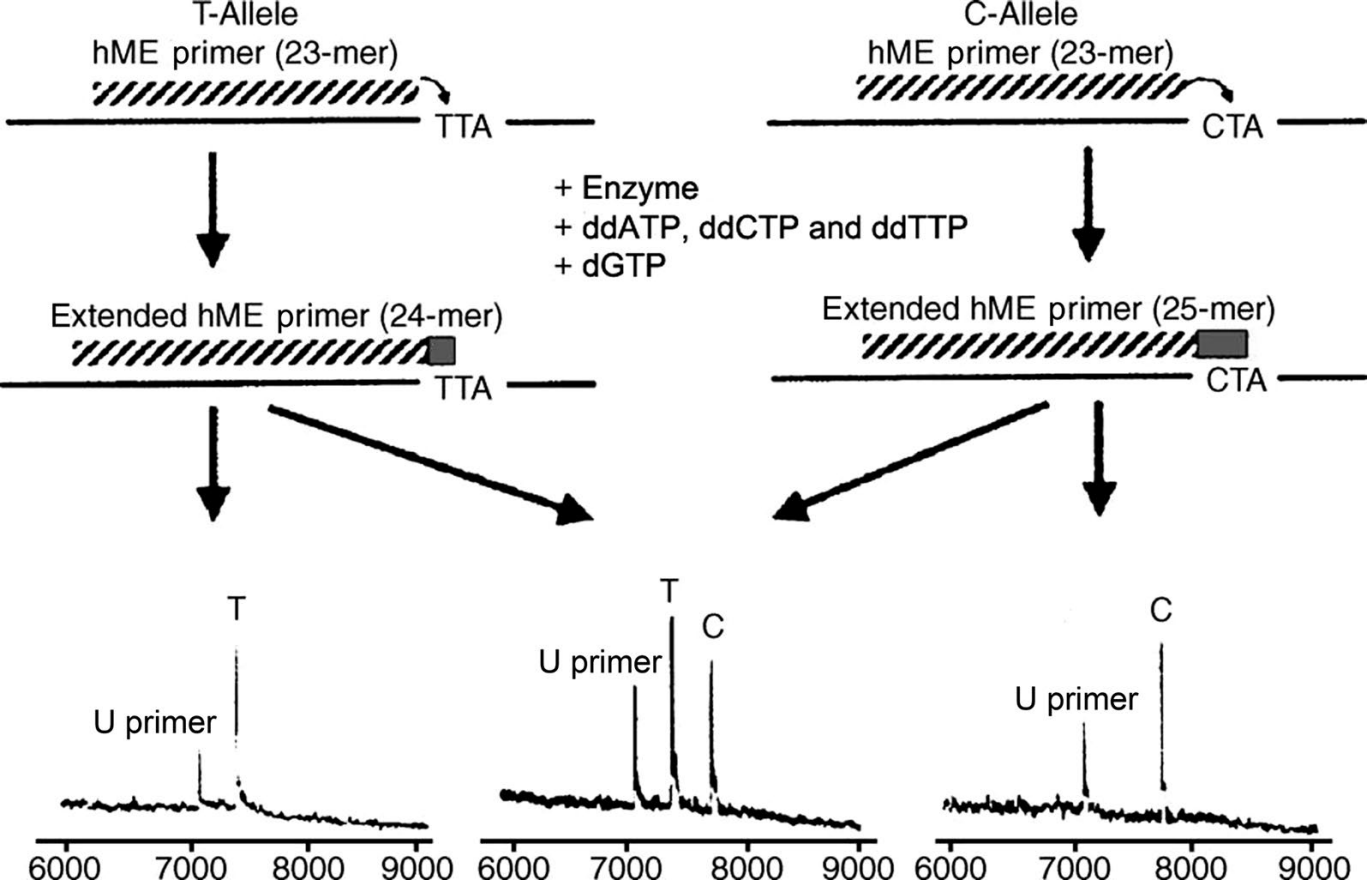
The homogeneous Mass EXTEND (hME) reaction. A deoxyguanosine triphosphate (dGTP) was used along with terminators dideoxyadenosine 5′-triphosphate (ddATP), dideoxycytidine 5′-triphosphate (ddCTP), and dideoxythymidine 5′-triphosphate (ddTTP). For the T allele (T), ddATP was incorporated, extending the primer to a 24-mer. For the C allele (C), dGTP was incorporated prior to the termination of the extension by incorporation of a ddATP, yielding a 25-mer. U primer marked unextended primer

Polymerase chain reaction (PCR) and extension primers were designed according to Assay Design 3.1 software (Sequenom Inc., San Diego, CA, USA) and synthesized by the Beijing Genomics Institute (Beijing, China). High-throughput MassARRAY spectrometry platform (Sequenom Inc.) was used for SNP genotyping. The homogeneous Mass EXTEND (hME) reaction is shown in Fig. 1. Following PCR amplification of a locus of interest, a primer extension was performed using an hME primer that was designed to anneal next to the SNP. The key feature of the scheme was the use of a terminator mixture that yielded allele-specific extension products differing in length and mass by at least one nucleotide. The genotyping was completed by Bomiao Biological Technology (Beijing, China). The polymorphisms genotyped are shown in Table 1. For quality control, we designed parallel group and blank group (DNA-free), and the reappear rate of the parallel group was 100%. Besides, all trial and analysis personnel were blinded to disease condition of all samples.

**Table 1:** The polymerase chain reaction (PCR) primers and homogeneous Mass EXTEND (hME) primers of 22 single nucleotide polymorphisms (SNPs) in three candidate genes

Gene	Localization	SNP	PCR primer (5′ → 3′)	hME primer (5′ → 3′)
*TYMS*	18p11.32	2790	First: ACGTTGGATGGGATGCCGAGGTAAAAGTTC	GATTTTTGACCTAGTTCCTT
			Second: ACGTTGGATGAACTGATAGGTCACGGACAG	
		2,853,741	First: ACGTTGGATGGGAAACAGATCTCAAACAGC	GGTACCACGTTTTCCTGCGGTCTTGTC
			Second: ACGTTGGATGAGCACAGTTCCCACGTTTTC	
		2,606,241	First: ACGTTGGATGCCACAGCTGAGAGTCTTAGG	GGGCGCAGTCCTTCCC
			Second: ACGTTGGATGACCAGACGGTTCCCAAAGG	
		3,786,362	First: ACGTTGGATGTTGGACAGCCTGGGATTCTC	GCCCAAGTCCCCTTC
			Second: ACGTTGGATGCAAAATGCCTCCACTGGAAG	
		1,004,474	First: ACGTTGGATGTAAAACTGTGACTCTCCCCC	GACCTCAGATGGTGATGTTCGTCTA
			Second: ACGTTGGATGGGGAAAGGCTGACATACATC	
		9,947,507	First: ACGTTGGATGAATTCTTCTGCCTCAGCCTC	GCCCCCGTCTCTACTAAAA
			Second: ACGTTGGATGTGGACAACATGGTGAAACCC	
		699,517	First: ACGTTGGATGCCACTGAAGAACCCTAAAAG	GGAGAAAGACTGACAATATCCTTC
			Second: ACGTTGGATGACTTTTACCTCGGCATCCAG	
		9,967,368	First: ACGTTGGATGTGGGTGACAGAGCCGTATG	AACCCAGATATTCCTTTCTATT
			Second: ACGTTGGATGGAATCCATGGTCTCCACAAC	
		15,872	First: ACGTTGGATGACAGAACTACACTACCAAGG	CCCCCCTCTCATGGTCACTGTTCC
			Second: ACGTTGGATGGAAAGTCCTCTCATGGTCAC	
*MTHFR*	1p36.3	1,801,133	First: ACGTTGGATGCTTGAAGGAGAAGGTGTCTG	AACGCGTGATGATGAAATCG
			Second: ACGTTGGATGTGCATGCCTTCACAAAGCGG	
		1,801,131	First: ACGTTGGATGTCTCCCGAGAGGTAAAGAAC	CATGAGCTGACCAGTGAAG
			Second: ACGTTGGATGAGGAGCTGCTGAAGATGTGG	
		3,737,964	First: ACGTTGGATGTGATGGCTGTAGATCCTCAC	GCAGCCCTCAAAAAAAACCTTTC
			Second: ACGTTGGATGTCAAATAGGAACCAGCCCTC	
		4,846,049	First: ACGTTGGATGAACTAAGCCCTCGAACCAAG	TGCACGGGCTCCAAG
			Second: ACGTTGGATGTGTTTTGCCTGTACTGCACG	
		2,274,976	First: ACGTTGGATGTATGTGTGTGTAGGACGAGG	CATACAGCTTTCCCCAC
			Second: ACGTTGGATGATGTACTGGATGATGGTGCG	
		3,753,582	First: ACGTTGGATGACGCAGTGGGCGCCAGGGA	CTCATTTTAAACCTGCCTCCCCGGCGA
			Second: ACGTTGGATGTGCCTTTTAAACCTGCCTCC	
		4,846,048	First: ACGTTGGATGTTTGGTTTGGTGGTGGCTTC	GGAATCAGTTAGTTCTGACACCAACAA
			Second: ACGTTGGATGTCCAGACCAGAAGCAGTTAG	
*RRM1*	11p15.5	725,519	First: ACGTTGGATGCACTTTAACTCTAGAAGATTG	TGGTGAAGAAATATGTAATGCCTCA
			Second: ACGTTGGATGGCCTAGCATATAAAGTGCTC	
		720,106	First: ACGTTGGATGGCAGTAATAAGAGCAGTTATC	AAACATTTATAACAAACTTAACATAC
			Second: ACGTTGGATGAAGGGTCAAATGAGTACCTC	
		1,042,858	First: ACGTTGGATGAGGGTTTGAAGACTGGGATG	GAACTGGATTGGATTAGC
			Second: ACGTTGGATGGCTTCTCCTTATTTAGAGTG	
		1,042,927	First: ACGTTGGATGCCACCAGTCAAAGCAGTAAA	GGTAAGTAAGGTTTCATCACCC
			Second: ACGTTGGATGCAGGGAGTGGTTAAGTAAGG	
		11,030,918	First: ACGTTGGATGTCCTGACGCAAATCAGAGCC	GGCTTACCCTGCCCTGCTTAAAAT
			Second: ACGTTGGATGCCACTGAAGAACCCTAAAAG	
		1,980,412	First: ACGTTGGATGGGTCTTCAGAACTATGAGAG	GGGCCAAAGGTATTTAAGTTTCCTATG
			Second: ACGTTGGATGCCAGAGGACAAAGGTATTTA	

*TYMS* thymidylate synthase, *MTHFR* methylene tetrahydrofolate reductase, *RRM1* ribonucleotide reductase M1

### Statistical analysis

Statistical analysis was performed using SPSS 19.0 and SNPStats softwares (IBM Inc., Chicago, IL, USA). The PLINK software was used to calculate genotype frequency, *P* value, and 95% confidence interval (CI). Hardy–Weinberg Equilibrium (HWE) was checked using the χ^2^ test or Fisher’s test. The loci deviated from HWE were excluded. The associations between polymorphisms and toxicity were estimated by unconditional logistic regression, and odds ratios (OR) and 95% CI were calculated. The ORs were adjusted for potential confounders, such as age and menstruation. All statistical tests were two-sided and considered significant when *P* was less than 0.05. Furthermore, we also examined the potential expression quantitative trait loci (eQTL) effects of the significant SNPs by extracting data from the Blood eQTL browser (https://www.genenetwork.nl/bloodeqtlbrowser/) [21].

## Results

### Patient characteristics

A total of 342 female patients with MBC were enrolled in our study, aging from 26 to 81 years (median, 51 years). All patients received capecitabine-based treatment, mostly in combination with docetaxel (59.9%) and vinorelbine (28.7%). The patient characteristics are summarized in Table 2. Of the 342 patients, 7 (2.0%) had complete response (CR), 194 (56.7%) had partial response (PR), 90 (26.3%) had stable disease (SD), 39 (11.4%) had progressive disease (PD), and 12 (3.5%) had unknown disease status; 141 (41.2%) experienced leukopenia, 109 (31.9%) had neutropenia, 101 (29.5%) had increased aminotransferase, 193 (56.4%) had HFS, 204 (59.6%) had nausea and vomiting, and 72 (21.1%) had increased bilirubin. HFS and increased bilirubin were relatively specific capecitabine-induced adverse events.

**Table 2:** Clinicopathological characteristics of 342 metastatic breast cancer patients (MBC) with or without hand-foot syndrome (HFS)

Characteristic	Total [cases (%)]	Patients with HFS [cases (%)]	Patients without HFS [cases (%)]
Age (years)			
≤ 40	57 (16.7)	30 (8.8)	27 (7.9)
> 40	285 (83.3)	163 (47.7)	122 (35.7)
Family history of cancer^a^			
None	275 (80.4)	152 (44.4)	123 (36.0)
Breast cancer or ovarian cancer	19 (5.6)	8 (2.3)	11 (3.2)
Other malignancies	48 (14.0)	33 (9.6)	15 (4.4)
Menstrual station			
Premenopause	218 (63.7)	120 (35.1)	98 (28.7)
Postmenopause	118 (34.5)	71 (20.8)	47 (13.7)
Unclear	6 (1.8)	2 (0.6)	4 (1.2)
Clinical stage			
I + II	154 (45.0)	89 (26.0)	65 (19.0)
III	143 (41.8)	85 (24.9)	58 (17.0)
IV	14 (4.1)	5 (1.5)	9 (2.6)
Unclear	31 (9.1)	14 (4.1)	17 (5.0)
Pathological type			
Intraductal carcinoma	4 (1.2)	2 (0.6)	2 (0.6)
Infiltrating ductal carcinoma	306 (89.5)	172 (50.3)	134 (39.2)
Invasive lobular carcinoma	17 (5.0)	11 (3.2)	6 (1.8)
Others	11 (3.2)	7 (2.0)	4 (1.2)
Unclear	4 (1.2)	1 (0.3)	3 (0.9)
Pathological grade			
Grade 1	10 (2.9)	8 (2.3)	2 (0.6)
Grade 2–3	170 (49.7)	96 (28.1)	74 (21.6)
Unclear	162 (47.4)	89 (26.0)	73 (21.3)
Vascular invasion			
No	308 (90.1)	174 (50.9)	134 (39.2)
Yes	34 (9.9)	19 (5.6)	15 (4.4)
Axillary lymph node metastasis at initial diagnosis			
No	106 (31.0)	54 (15.8)	52 (15.2)
Yes	225 (65.8)	136 (39.8)	89 (26.0)
Unclear	11 (3.2)	3 (0.9)	8 (2.3)
Distant metastasis at initial diagnosis			
No	324 (94.7)	186 (54.4)	138 (40.4)
Yes	14 (4.1)	5 (1.5)	9 (2.6)
Unclear	4 (1.2)	2 (0.6)	2 (0.6)
ER status			
Positive	215 (62.9)	123 (36.0)	92 (26.9)
Negative	117 (34.2)	65 (19.0)	52 (15.2)
Unclear	10 (2.9)	5 (1.5)	5 (1.5)
PR status			
Positive	213 (62.3)	117 (34.2)	96 (28.1)
Negative	118 (34.5)	70 (20.5)	48 (14.0)
Unclear	11 (3.2)	6 (1.8)	5 (1.5)
HER2 status			
Positive	83 (24.3)	47 (13.7)	36 (10.5)
Negative	230 (67.3)	130 (38.0)	100 (29.2)
Unclear^b^	29 (8.5)	16 (4.7)	13 (3.8)
Therapy			
Capecitabine	20 (5.8)	10 (2.9)	10 (2.9)
Docetaxel plus capecitabine	205 (59.9)	123 (36.0)	82 (24.0)
Vinorelbine plus capecitabine	98 (28.7)	48 (14.0)	50 (14.6)
Others^c^	19 (5.6)	12 (3.5)	7 (2.0)
Therapy line			
First-line	211 (61.7)	121 (35.4)	90 (26.3)
Multi-line	131 (38.3)	72 (21.1)	59 (17.3)
Metastatic site			
No visceral metastasis (including local recurrence)	104 (30.4)	60 (17.5)	44 (12.9)
Visceral metastasis	238 (69.6)	133 (38.9)	105 (30.7)
Maintenance therapy			
Yes	137 (40.1)	94 (27.5)	43 (12.6)
No	205 (59.9)	99 (28.9)	106 (31.0)
Response evaluation			
CR	7 (2.0)	5 (1.5)	2 (0.6)
PR	194 (56.7)	107 (31.3)	87 (25.4)
SD	90 (26.3)	56 (16.4)	34 (9.9)
PD	39 (11.4)	20 (5.8)	19 (5.6)
Unclear	12 (3.5)	5 (1.5)	7 (2.0)
Survival condition^d^			
Alive	218 (63.7)	124 (36.3)	94 (27.5)
Dead	124 (36.3)	69 (20.2)	55 (16.1)

*ER* estrogen receptor, *PR* progesterone receptor, *HER2* human epidermal growth factor receptor 2, *CR* complete response, *PR* partial response, *SD* stable disease, *PD* progressive disease

^a^Breast cancer or ovarian cancer or other malignances of first- or second-degree relatives

^b^Equivocal results (HER2++) without fluorescence in situ hybridization testing

^c^Other capecitabine-based therapies

^d^Patients’ survival conditions by last follow-up

### Associations between gene polymorphisms and risks of increased bilirubin and HFS

No association between polymorphisms of the 3 candidate genes and the risk of increased bilirubin was found (data not shown). There was no significant association between polymorphisms of *RRM1* and the risk of HFS (Table 3). The most investigated polymorphisms of *MTHFR*, rs1801133 and rs1801131, were not related with 5-FU toxicities in our study. We found that *TYMS* rs2606241 (*P *= 0.022), *TYMS* rs2853741 (*P *= 0.019), *MTHFR* rs3737964 (*P *= 0.029), and *MTHFR* rs4846048 (*P *= 0.030) were associated with the risk of HFS (Table 3).

**Table 3:** Frequencies of 22 SNPs of 3 genes in metastatic breast cancer patients with and without capecitabine-induced HFS

Gene	SNP	Genotype	Patients with HFS [cases (%)]	Patients without HFS [cases (%)]	*P* value
Total			193	149	
*TYMS*	rs2790	AA	78 (40.4)	48 (32.2)	0.316
		GA	87 (45.1)	74 (49.7)	
		GG	26 (13.5)	24 (16.1)	
		NA	2 (1.0)	3 (2.0)	
	rs15872	TT	73 (37.8)	68 (45.6)	0.265
		TC	96 (49.7)	61 (40.9)	
		CC	22 (11.4)	17 (11.4)	
		NA	2 (1.0)	3 (2.0)	
	rs699517	TT	73 (37.8)	70 (47.0)	0.195
		TC	97 (50.3)	61 (40.9)	
		CC	20 (10.4)	15 (10.1)	
		NA	3 (1.6)	3 (2.0)	
	rs1004474	AA	61 (31.6)	45 (30.2)	0.366
		GA	97 (50.3)	69 (46.3)	
		GG	32 (16.6)	34 (22.8)	
		NA	3 (1.6)	1 (0.7)	
	rs2606241	GG	44 (22.8)	34 (22.8)	*0.022*
		GT	113 (58.5)	69 (46.3)	
		TT	33 (17.1)	44 (29.5)	
		NA	3 (1.6)	2 (1.3)	
	rs2853741	TT	38 (19.7)	48 (32.2)	*0.019*
		TC	113 (58.5)	68 (45.6)	
		CC	39 (20.2)	31 (20.8)	
		NA	3 (1.6)	2 (1.3)	
	rs3786362	TT	135 (69.9)	86 (57.7)	0.055
		CT	48 (24.9)	53 (35.6)	
		CC	7 (3.6)	8 (5.4)	
		NA	3 (1.6)	2 (1.3)	
	rs9947507	TT	193 (100.0)	149 (100.0)	NA
	rs9967368	CC	55 (28.5)	52 (34.9)	0.231
		CG	97 (50.3)	61 (40.9)	
		GG	38 (19.7)	33 (22.1)	
		NA	3 (1.6)	3 (2.0)	
*MTHFR*	rs1801131	AA	143 (74.1)	101 (67.8)	0.291
		CA	47 (24.4)	45 (30.2)	
		CC	1 (0.5)	1 (0.7)	
		NA	2 (1.0)	2 (1.3)	
	rs1801133	TT	60 (31.1)	52 (34.9)	0.662
		TC	99 (51.3)	69 (46.3)	
		CC	31 (16.1)	25 (16.8)	
		NA	3 (1.6)	3 (2.0)	
	rs2274976	GG	163 (84.5)	131 (87.9)	0.558
		GA	26 (13.5)	17 (11.4)	
		AA	1 (0.5)	0 (0.0)	
		NA	3 (1.6)	1 (0.7)	
	rs3737964	GG	165 (85.5)	113 (75.8)	*0.029*
		AG	25 (13.0)	33 (22.1)	
		NA	3 (1.6)	3 (2.0)	
	rs3753582	TT	160 (82.9)	131 (87.9)	0.389
		GT	29 (15.0)	15 (10.1)	
		GG	1 (0.5)	1 (0.7)	
		NA	3 (1.6)	2 (1.3)	
	rs4846048	AA	165 (85.5)	115 (77.2)	*0.030*
		AG	25 (13.0)	33 (22.1)	
		NA	3 (1.6)	1 (0.7)	
	rs4846049	GG	138 (71.5)	97 (65.1)	0.338
		GT	49 (25.4)	49 (32.9)	
		TT	3 (1.6)	2 (1.3)	
		NA	3 (1.6)	1 (0.7)	
*RRM1*	rs720106	AA	142 (73.6)	98 (65.8)	0.054
		AG	40 (20.7)	46 (30.9)	
		GG	7 (3.6)	2 (1.3)	
		NA	4 (2.1)	3 (2.0)	
	rs725519	AA	142 (73.6)	98 (65.8)	0.532
		AG	40 (20.7)	46 (30.9)	
		GG	7 (3.6)	2 (1.3)	
		NA	4 (2.1)	3 (2.0)	
	rs1042858	AA	116 (60.1)	78 (52.3)	0.491
		CA	64 (33.2)	56 (37.6)	
		CC	10 (5.2)	9 (6.0)	
		NA	3 (1.6)	6 (4.0)	
	rs1042927	AA	118 (61.1)	81 (54.4)	0.415
		CA	61 (31.6)	57 (38.3)	
		CC	11 (5.6)	9 (6.0)	
		NA	3 (1.6)	2 (1.3)	
	rs1980412	CC	61 (31.6)	58 (38.9)	0.359
		TC	102 (52.8)	69 (46.3)	
		TT	26 (13.5)	19 (12.8)	
		NA	4 (2.1)	3 (2.0)	
	rs11030918	TT	103 (53.4)	74 (49.7)	0.378
		CT	66 (34.2)	61 (40.9)	
		CC	21 (10.9)	12 (8.1)	
		NA	3 (1.6)	2 (1.3)	

*P* value < 0.05 was considered statistically significant (in italics)

SNP, single nucleotide polymorphism; HFS, hand-foot syndrome; *TYMS*, thymidylate synthase; *MTHFR*, methylene tetrahydrofolate reductase; *RRM1*, ribonucleotide reductase M1; NA, not applicable

We performed unconditional logistic regression analysis using the SNPStats software. The results indicated that the genotype AG of *MTHFR* rs3737964 (OR = 0.54, 95% CI 0.31–0.97, *P *= 0.038) and *MTHFR* rs4846048 (OR = 0.54, 95% CI 0.30–0.98, *P* = 0.042) were protective factors for HFS, whereas the genotype CT of *TYMS* rs2853741 (OR = 2.25, 95% CI 1.31–3.87, *P* = 0.012) was a risk factor for HFS (Table 4). However, the association between the genotype GT of *TYMS* rs2606241 (OR = 1.27, 95% CI 0.73–2.23, *P* = 0.012) and HFS was still uncertain.

**Table 4:** Unconditional logistic regression analyses assessing associations of gene polymorphisms with HFS

Gene	SNP	Genetic model	Genotype	Patients without HFS [cases (%)]	Patients with HFS [cases (%)]	OR (95% CI)	*P* value	AIC	BIC
*TYMS*	rs2606241	Codominant	GG	34 (22.8)	44 (22.8)	1.00	0.012	449.7	476.4
			GT	69 (46.3)	113 (58.5)	1.27 (0.73–2.23)			
			TT	44 (29.5)	33 (17.1)	0.55 (0.28–1.06)			
		Dominant	GG	34 (22.8)	44 (22.8)	1.00	0.960	456.5	479.4
			GT + TT	113 (75.8)	146 (75.6)	0.99 (0.58–1.68)			
		Recessive	GG + GT	103 (69.1)	157 (81.3)	1.00	0.004	448.4	471.3
			TT	44 (29.5)	33 (17.1)	0.46 (0.27–0.79)			
		Overdominant	GG + TT	78 (52.3)	77 (39.9)	1.00	0.018	450.9	473.8
			GT	69 (46.3)	113 (58.5)	1.72 (1.10–2.70)			
		Log-additive	NA	NA	NA	0.74 (0.53–1.03)	0.071	453.3	476.2
	rs2853741	Codominant	TT	48 (32.2)	38 (19.9)	1.00	0.012	449.6	476.4
			CT	68 (45.6)	113 (58.5)	2.25 (1.31–3.87)			
			CC	31 (20.8)	39 (20.2)	1.71 (0.81–3.31)			
		Dominant	TT	48 (32.2)	38 (19.9)	1.00	0.005	448.5	471.4
			TC + CC	99 (66.4)	152 (78.8)	2.09 (1.25–3.49)			
		Recessive	TT + TC	116 (77.9)	151 (78.2)	1.00	0.950	456.4	479.4
			CC	31 (20.8)	39 (20.2)	0.98 (0.57–1.70)			
		Overdominant	TT + CC	79 (53.0)	77 (38.9)	1.00	0.012	450.1	473.1
			TC	68 (45.6)	113 (58.5)	1.77 (1.13–2.77)			
		Log-additive	NA	NA	NA	1.35 (0.97–1.88)	0.075	453.3	476.2
*MTHFR*	rs4846048	NA	AA	115 (77.2)	165 (85.5)	1.00	0.042	453.7	476.7
			AG	33 (22.1)	25 (13.0)	0.54 (0.30–0.98)			
	rs3737964	NA	GG	113 (75.8)	165 (85.5)	1.00	0.038	458.8	470.3
			AG	33 (22.1)	25 (13.0)	0.54 (0.31–0.97)			

The lower the AIC and BIC values, the more accurate the model

*HFS* hand-food syndrome, *OR* odds ratio, *CI* confidence interval, *AIC* Akaike’s information criterion, *BIC* Bayes information criterion, *NA* not applicable

### Associations between the SNPs and corresponding gene expression

*TYMS* rs2606241 and rs2853741 were not associated with *TYMS* expression, whereas *MTHFR* rs3737964 and rs4846048 were significantly associated with *MTHFR* expression (both *P *< 0.001).

## Discussion

The present study demonstrated that genetic polymorphisms in *MTHFR* and *TYMS* were associated with capecitabine-induced HFS in MBC patients. We identified 4 SNPs significantly associated with HFS. Further analysis showed that the genotype AG of *MTHFR* rs3737964 and the genotype AG of *MTHFR* rs4846048 were protective factors for HFS, whereas the genotype CT of *TYMS* rs2853741 was a risk factor.

Until recently, few researches have explored the biomarkers for toxicity of capecitabine in patients with MBC. Two retrospective studies [22, 23] and an exploratory analysis [24] have shown that HFS occurrence in capecitabine-treated patients might be associated with improved efficacy and suggested that early dose adjustment according to the severity of HFS might improve its efficacy. However, there is still little information available about predictive biomarkers for HFS. rs9936750, a polymorphism in an intergenic region of different genes, was shown to be associated with an increased risk of capecitabine-induced HFS [25], but the sample size is too small to be convincing and the result needs further confirmation. Another variant rs3215400 in the cytidine deaminase promoter has been demonstrated to be plausibly associated with severe capecitabine-induced HFS [18], but such association was not observed in another study [26]. In the present study, we identified 4 potential predictive biomarkers for HFS in Chinese female MBC patients treated with capecitabine, so that capecitabine therapy might be further tailored to patient response.

**Fig. 2:**
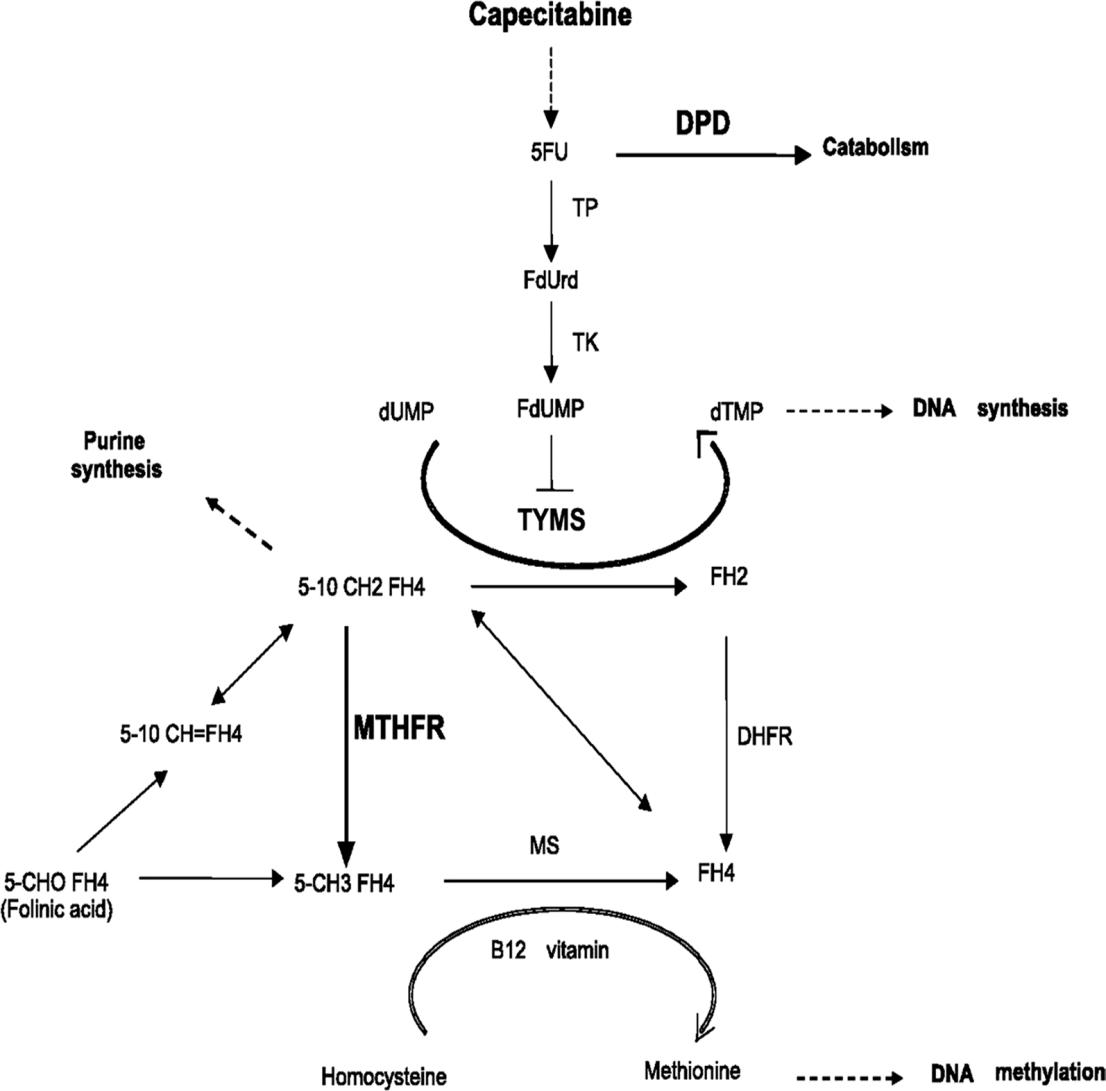
Metabolic pathways of capecitabine and pharmacologically related targets. *5-FU* 5-fluorouracil, *DPD* dihydropyrimidine dehydrogenase, *TP* thymidine phosphorylase, *FdUrd* 5-fluorodeoxyuridine, *TK* thymidine kinase, *dUMP* deoxyuridine monophosphate, *FdUMP* fluorodeoxyuridine 5′-monophosphate, *dTMP* deoxythymidine monophosphate, *DNA* deoxyribonucleic acid, *TYMS* thymidylate synthase, *5-10 CH2FH4* 5-10 methylene-tetrahydrofolate, *FH2* dihydrofolate, *5-10 CH=FH4* 5-10 methenyltetrahydrofolate, *MTHFR* methylenetetrahydrofolate reductase, *DHFR* dihydrofolate reductase, *5-CHOFH4* 5-formyltetrahydrofolate, *5-CH3FH4* 5-methyltetrahydrofolate, *MS* methionine synthase, *FH4* tetrahydrofolate

Optimal efficacy of 5-FU requires elevated intratumoral concentration of 5-10 MTHF, which is mainly controlled by MTHFR [27]. As 5-10 MTHF inhibits TYMS activity in conjunction with 5-fluorodeoxyuridine 5′-monophosphate (5-FdUMP), reduced *MTHFR* activity, which is associated with increased levels of 5-10 MTHF, theoretically leads to more effective TYMS inhibition. The metabolic pathway of capecitabine is shown in Fig. 2. Previous clinical studies have suggested that *MTHFR* gene polymorphisms might have impact on fluoropyrimidine responsiveness [28, 29]. Two of the most investigated polymorphisms of *MTHFR*, rs1801133 (Ala222Val, 677C>T) and rs1801131 (Glu429Ala, 1298A>C), have been proved to have close association with the onset of some cancers, including colorectal cancer [30] and breast cancer [31]. Several meta-analyses have demonstrated that *MTHFR C677T* polymorphism may be a risk factor for thyroid [32], breast and ovarian cancers [33]. Accordingly, we investigated influence of *MTHFR* rs1801133 and rs1801131 on toxicities of capecitabine therapy in patients with MBC, but found no such associations. This result was in agreement with those of some [34–36], whereas other studies have demonstrated significant associations between *MTHFR* rs1801133 and 5-FU-induced toxicity [37, 38]. The reason for this discrepancy is not clear. It may be due to different sample sizes or patient selection. Large-scale studies are needed to determine whether testing for these two variants is clinically useful. Our results showed that the frequencies of the genotype AG of *MTHFR* rs4846048 (*P* = 0.030) and *MTHFR* rs3737964 (*P* = 0.029) were significantly lower in patients with capecitabine-induced HFS than in those without HFS. Further multivariate unconditional logistic regression analysis indicated that the genotype AG of *MTHFR* rs3737964 (OR = 0.54, 95% CI 0.31–0.97, *P* = 0.038) and *MTHFR* rs4846048 (OR = 0.54, 95% CI 0.30–0.98, *P* = 0.042) were protective factors for HFS. Due to the catalytic deficit of MTHFR, subsequent to its polymorphic variants, the increased 5-10 MTHF concentration enhanced the formation and stability of the inhibitory complex composing of 5-10 MTHF, TYMS, and 5-FdUMP, thereby increasing the potential toxicity of fluoropyrimidines [39]. Lying in the promoter region of *MTHFR*, rs3737964 did not result in coding amino acid polymorphisms, but possibly led to transcription factor binding difference. rs4846048, located on the 3′-untranslated region (3′-UTR) of *MTHFR*, might influence the microRNA (miRNA) binding regulation, thus enhancing the message RNA (mRNA) expression. Further eQTL analyses based on the Blood eQTL browser confirmed that the alleles of rs3737964 and rs4846048 could affect the gene expression levels of *MTHFR* in *cis*. Therefore, it was inferred that the MTHFR enzymatic activity in subjects with the genotype AG of rs3737964 and rs4846048 might be improved through increasing mRNA transcription, and hence to protect patients from HFS.

TYMS is an enzyme that catalyzes the conversion of dUMP to dTMP, and is the main intracellular target of the active 5-FU metabolite, 5-FdUMP, which forms a ternary complex with TYMS and 5-10 MTHF [40]. Elevated TYMS expression or activity is a well-known mechanism of resistance to 5-FU [41]. Ooyama et al. [42] found that the copy number of *TYMS* (18p11.32) showed a strong association with drug resistance, which may lead to the use of *TYMS* copy number as a predictive marker for drug sensitivity of fluoropymidines. The rs2612091 and rs2741171 variants, lying downstream of *TYMS* within an intron of enolase superfamily member 1 (*ENOSF1*), were significant associated with HFS, irrespective of the two *TYMS* polymorphisms {5′-variable number of tandem repeat (5′-VNTR) 2R/3R [36] and 3′-UTR 6 bp ins-del [43]} that have previously been reported to affect 5-FU toxicity [16]. Therefore, *TYMS* rs2612091 and rs2741171 were not included in the present study. We included 9 less-investigated polymorphisms of *TYMS* and found differences in frequencies of *TYMS* rs2606241 and rs2853741 between patients with and without HFS. Our results showed that the frequencies of the genotype GT of *TYMS* rs2606241 (*P* = 0.022) and the genotype CT of *TYMS* rs2853741 (*P* = 0.019) were significantly higher in patients with capecitabine-induced HFS than in those without HFS. Logistic regression reflected that the genotype CT of *TYMS* rs2853741 (OR = 2.25, 95% CI 1.31–3.87, *P* = 0.012) seemed to raise the susceptibility to HFS. Although the association was uncertain, the genotype GT of *TYMS* rs2606241 (OR = 1.27, 95% CI 0.73–2.23, *P* = 0.012) tended to increase the risk of HFS. Lecomte et al. [43] suggested that the low *TYMS* mRNA expression level in patients with 2R/2R genotype was associated with a higher risk of 5-FU-induced adverse events. Falling both in the promoter region of *TYMS*, the genotype GT of rs2606241 and the genotype CT of rs2853741 did not change the coding amino acid sequence. However, the mRNA expression might be reduced due to the impact on the transcription binding sites. Therefore, we hypothesized that the decreasing *TYMS* mRNA expression in patients with the genotype GT of rs2606241 and the genotype CT of rs2853741 might increase the risk of 5-FU-induced HFS because of the high efficacy of TYMS inhibition. However, further eQTL data did not afford much information about the association between rs2606241 and rs2853741 and gene expression level of *TYMS*. Whether these two polymorphisms are implicated in gene regulation at a post-transcriptional level through decreased mRNA stability needs further validation.

The specific mechanisms underlying the effect of the above mentioned genotypes on HFS are still not quite valid. We also cannot neglect that these results must be view as preliminary and need additional confirmation. Further prospective studies on a larger number of patients are desirable to confirm and quantitate these associations in additional datasets and to understand the mechanistic origins of capecitabine toxicity. Besides, further efforts to identify additional polymorphisms and rare variants associated with capecitabine toxicity remain valid.

## Conclusions

In summary, we have identified a panel of potentially useful pharmacogenetic markers predicting capecitabine-induced HFS in MBC patients. Our findings may help clinicians identify patients who have a low risk of capecitabine-induced HFS and improve treatment decision for MBC patients.

## Data Availability

The data used to support the findings of this study are available from the corresponding author upon request.

## Abbreviations

MBC: metastatic breast cancer
HFS: hand-foot syndrome
5-FU: 5-fluorouracil
TYMS: thymidylate synthase
MTHFR: methylene tetrahydrofolate reductase
RRM1: ribonucleotide reductase M1
dUMP: deoxyuridine monophosphate
dTMP: deoxythymidine monophosphate
5-10 MTHF: 5-10 methylenetetrahydrofolate
5-MeTHF: 5-methylenetetrahydrofolate
FUDP: fluorouridine diphosphate
FdUDP: fluorodeosyuridine diphosphate
SNP: single nucleotide polymorphism
DPYD: dehydrogenase gene
CDD: cytidine deaminase gene
ER: estrogen receptor
PR: progesterone receptor
HER2: human epidermal growth factor receptor 2
PCR: polymerase chain reaction
hME: homogeneous Mass EXTEND
CI: confidence interval
OR: odds ratios
eQTL: expression quantitative trait loci
CR: complete response
PR: partial response
SD: stable disease
PD: progressive disease
3′-UTR: 3′-untranslated region
miRNA: microRNA
ENOSF1: enolase superfamily member 1
5′-VNTR: 5′-variable number of tandem repeat
DPD: dihydropyrimidine dehydrogenase
TP: thymidine phosphorylase
FdUrd: 5-fluorodeoxyuridine
TK: thymidine kinase
FdUMP: fluorodeoxyuridine 5′-monophosphate
5-10 CH2 FH4: 5-10 methylene-tetrahydrofolate
FH2: dihydrofolate
5-10 CH=FH4: 5-10 methenyltetrahydrofolate
DHFR: dihydrofolate reductase
5-CHO FH4: 5-formyltetrahydrofolate
5-CH3 FH4: 5-methyltetrahydrofolate
MS: methionine synthase
FH4: tetrahydrofolate

## References

[1] da Costa Vieira RA, Biller G, Uemura G, Ruiz CA, Curado MP (2017). Breast cancer screening in developing countries. Clinics.

[2] Dawood S, Broglio K, Ensor J, Hortobagyi GN, Giordano SH (2010). Survival differences among women with de novo stage IV and relapsed breast cancer. Ann Oncol.

[3] Cetin B, Benekli M, Turker I, Koral L, Ulas A, Dane F (2014). Lapatinib plus capecitabine for HER2-positive advanced breast cancer: a multicentre study of Anatolian Society of Medical Oncology (ASMO). J Chemother.

[4] Saura C, Garcia-Saenz JA, Xu B, Harb W, Moroose R, Pluard T (2014). Safety and efficacy of neratinib in combination with capecitabine in patients with metastatic human epidermal growth factor receptor 2-positive breast cancer. J Clin Oncol.

[5] Schmoll HJ, Twelves C, Sun W, O’Connell MJ, Cartwright T, McKenna E (2014). Effect of adjuvant capecitabine or fluorouracil, with or without oxaliplatin, on survival outcomes in stage III colon cancer and the effect of oxaliplatin on post-relapse survival: a pooled analysis of individual patient data from four randomised controlled trials. Lancet Oncol..

[6] Bang YJ, Kim YW, Yang HK, Chung HC, Park YK, Lee KH (2012). Adjuvant capecitabine and oxaliplatin for gastric cancer after D2 gastrectomy (CLASSIC): a phase 3 open-label, randomised controlled trial. Lancet.

[7] Brandi G, Venturi M, De Lorenzo S, Garuti F, Frega G, Palloni A (2018). Sustained complete response of advanced hepatocellular carcinoma with metronomic capecitabine: a report of three cases. Cancer Commun.

[8] Yu X, Wang QX, Xiao WW, Chang H, Zeng ZF, Lu ZH (2018). Neoadjuvant oxaliplatin and capecitabine combined with bevacizumab plus radiotherapy for locally advanced rectal cancer: results of a single-institute phase II study. Cancer Commun.

[9] Pallis AG, Boukovinas I, Ardavanis A, Varthalitis I, Malamos N, Georgoulias V (2012). A multicenter randomized phase III trial of vinorelbine/gemcitabine doublet versus capecitabine monotherapy in anthracycline- and taxane-pretreated women with metastatic breast cancer. Ann Oncol.

[10] Baratelli C, Zichi C, Di Maio M, Brizzi MP, Sonetto C, Scagliotti GV (2018). A systematic review of the safety profile of the different combinations of fluoropyrimidines and oxaliplatin in the treatment of colorectal cancer patients. Crit Rev Oncol Hematol.

[11] Peng J, Dong C, Wang C, Li W, Yu H, Zhang M (2018). Cardiotoxicity of 5-fluorouracil and capecitabine in Chinese patients: a prospective study. Cancer Commun.

[12] Webster-Gandy JD, How C, Harrold K (2007). Palmar-plantar erythrodysesthesia (PPE): a literature review with commentary on experience in a cancer centre. Eur J Oncol Nurs..

[13] Miwa M, Ura M, Nishida M, Sawada N, Ishikawa T, Mori K (1998). Design of a novel oral fluoropyrimidine carbamate, capecitabine, which generates 5-fluorouracil selectively in tumours by enzymes concentrated in human liver and cancer tissue. Eur J Cancer.

[14] Thorn CF, Marsh S, Carrillo MW, McLeod HL, Klein TE, Altman RB (2011). PharmGKB summary: fluoropyrimidine pathways. Pharmacogenet Genomics.

[15] Xu XL, Zheng J, Mao WM, Ling ZQ (2016). RRM1 *151A>T, RRM1 −756T>C, and RRM1 −585T>Gis associated with increased susceptibility of lung cancer in Chinese patients. Cancer Med.

[16] Rosmarin D, Palles C, Pagnamenta A, Kaur K, Pita G, Martin M (2015). A candidate gene study of capecitabine-related toxicity in colorectal cancer identifies new toxicity variants at DPYD and a putative role for ENOSF1 rather than TYMS. Gut.

[17] Rosmarin D, Palles C, Church D, Domingo E, Jones A, Johnstone E (2014). Genetic markers of toxicity from capecitabine and other fluorouracil-based regimens: investigation in the QUASAR2 study, systematic review, and meta-analysis. J Clin Oncol.

[18] Caronia D, Martin M, Sastre J, de la Torre J, Garcia-Saenz JA, Alonso MR (2011). A polymorphism in the cytidine deaminase promoter predicts severe capecitabine-induced hand-foot syndrome. Clin Cancer Res.

[19] Loganayagam A, Arenas Hernandez M, Corrigan A, Fairbanks L, Lewis CM, Harper P (2013). Pharmacogenetic variants in the DPYD, TYMS, CDA and MTHFR genes are clinically significant predictors of fluoropyrimidine toxicity. Br J Cancer.

[20] Abecasis GR, Altshuler D, Auton A, Brooks LD, Durbin RM, Gibbs RA (2010). A map of human genome variation from population-scale sequencing. Nature.

[21] Westra H-J, Peters MJ, Esko T, Yaghootkar H, Schurmann C, Kettunen J (2013). Systematic identification of trans eQTLs as putative drivers of known disease associations. Nat Genet.

[22] Azuma Y, Hata K, Sai K, Udagawa R, Hirakawa A, Tohkin M (2012). Significant association between hand-foot syndrome and efficacy of capecitabine in patients with metastatic breast cancer. Biol Pharm Bull.

[23] Kurt M, Aksoy S, Guler N (2006). Could the hand-foot syndrome after capecitabine treatment be associated with better outcome in metastatic breast cancer patients?. Acta Oncol.

[24] Zielinski C, Lang I, Beslija S, Kahan Z, Inbar MJ, Stemmer SM (2016). Predictive role of hand-foot syndrome in patients receiving first-line capecitabine plus bevacizumab for HER2-negative metastatic breast cancer. Br J Cancer.

[25] Wheeler HE, Gonzalez-Neira A, Pita G, de la Torre-Montero JC, Alonso R, Lopez-Fernandez LA (2014). Identification of genetic variants associated with capecitabine-induced hand-foot syndrome through integration of patient and cell line genomic analyses. Pharmacogenet Genomics.

[26] Ribelles N, Lopez-Siles J, Sanchez A, Gonzalez E, Sanchez MJ, Carabantes F (2008). A carboxylesterase 2 gene polymorphism as predictor of capecitabine on response and time to progression. Curr Drug Metab.

[27] Largillier R, Etienne-Grimaldi MC, Formento JL, Ciccolini J, Nebbia JF, Ginot A (2006). Pharmacogenetics of capecitabine in advanced breast cancer patients. Clin Cancer Res.

[28] Cohen V, Panet-Raymond V, Sabbaghian N, Morin I, Batist G, Rozen R (2003). Methylenetetrahydrofolate reductase polymorphism in advanced colorectal cancer: a novel genomic predictor of clinical response to fluoropyrimidine-based chemotherapy. Clin Cancer Res.

[29] Etienne MC, Formento JL, Chazal M, Francoual M, Magne N, Formento P (2004). Methylenetetrahydrofolate reductase gene polymorphisms and response to fluorouracil-based treatment in advanced colorectal cancer patients. Pharmacogenetics..

[30] Xie SZ, Liu ZZ, Yu JH, Liu L, Wang W, Xie DL (2015). Association between the MTHFR C677T polymorphism and risk of cancer: evidence from 446 case-control studies. Tumour Biol.

[31] Li K, Li W, Dong X (2014). Association of 677 C>T (rs1801133) and 1298 A>C (rs1801131) polymorphisms in the MTHFR gene and breast cancer susceptibility: a meta-analysis based on 57 individual studies. PLoS ONE.

[32] Yan Y, Han F, Fu H, Xia W, Qin X (2014). Association between MTHFR C677T polymorphism and thyroid cancer risk: a meta-analysis. Tumour Biol.

[33] He L, Shen Y (2017). MTHFR C677T polymorphism and breast, ovarian cancer risk: a meta-analysis of 19,260 patients and 26,364 controls. Onco Targets Ther..

[34] Ruzzo A, Graziano F, Loupakis F, Rulli E, Canestrari E, Santini D (2007). Pharmacogenetic profiling in patients with advanced colorectal cancer treated with first-line FOLFOX-4 chemotherapy. J Clin Oncol.

[35] Ruzzo A, Graziano F, Loupakis F, Santini D, Catalano V, Bisonni R (2008). Pharmacogenetic profiling in patients with advanced colorectal cancer treated with first-line FOLFIRI chemotherapy. Pharmacogenomics J..

[36] Schwab M, Zanger UM, Marx C, Schaeffeler E, Klein K, Dippon J (2008). Role of genetic and nongenetic factors for fluorouracil treatment-related severe toxicity: a prospective clinical trial by the German 5-FU Toxicity Study Group. J Clin Oncol.

[37] Gusella M, Frigo AC, Bolzonella C, Marinelli R, Barile C, Bononi A (2009). Predictors of survival and toxicity in patients on adjuvant therapy with 5-fluorouracil for colorectal cancer. Br J Cancer.

[38] Afzal S, Jensen SA, Vainer B, Vogel U, Matsen JP, Sorensen JB (2009). MTHFR polymorphisms and 5-FU-based adjuvant chemotherapy in colorectal cancer. Ann Oncol.

[39] Longley DB, Harkin DP, Johnston PG (2003). 5-fluorouracil: mechanisms of action and clinical strategies. Nat Rev Cancer.

[40] Pinedo HM, Peters GF (1988). Fluorouracil: biochemistry and pharmacology. J Clin Oncol.

[41] Etienne MC, Chazal M, Laurent-Puig P, Magne N, Rosty C, Formento JL (2002). Prognostic value of tumoral thymidylate synthase and p53 in metastatic colorectal cancer patients receiving fluorouracil-based chemotherapy: phenotypic and genotypic analyses. J Clin Oncol.

[42] Ooyama A, Okayama Y, Takechi T, Sugimoto Y, Oka T, Fukushima M (2007). Genome-wide screening of loci associated with drug resistance to 5-fluorouracil-based drugs. Cancer Sci.

[43] Lecomte T, Ferraz JM, Zinzindohoue F, Loriot MA, Tregouet DA, Landi B (2004). Thymidylate synthase gene polymorphism predicts toxicity in colorectal cancer patients receiving 5-fluorouracil-based chemotherapy. Clin Cancer Res.

